# Progesterone Aggravates Lung Fibrosis in a Mouse Model of Systemic Sclerosis

**DOI:** 10.3389/fimmu.2021.742227

**Published:** 2021-11-29

**Authors:** Fatemeh Vafashoar, Kazem Mousavizadeh, Hadi Poormoghim, Amir Haghighi, Salar Pashangzadeh, Nazanin Mojtabavi

**Affiliations:** ^1^ Institute of Immunology and Infectious Disease, Immunology Research Center, Iran University of Medical Sciences, Tehran, Iran; ^2^ Department of Immunology, Iran University of Medical Sciences, Tehran, Iran; ^3^ Department of Pharmacology, School of Medicine, Iran University of Medical Sciences, Tehran, Iran; ^4^ Scleroderma Study Group, Firuzgar Hospital, Iran University of Medical Sciences, Tehran, Iran; ^5^ Ludwik Rydygier Collegium Medicum in Bydgoszcz, Nicolaus Copernicus University in Torun, Bydgoszcz, Poland

**Keywords:** progesterone, systemic sclerosis, scleroderma, lung, fibrosis, bleomycin

## Abstract

**Background:**

Gender-related factors have explained the higher prevalence of autoimmune diseases in women. Sex hormones play a key role in the immune system and parenchymal cells function; therefore, these hormones can be important in the pathogenesis of autoimmune diseases as a risk or beneficial factor. Lung fibrosis is the main cause of mortality in systemic sclerosis, a female predominant autoimmune disease. The objective of this study was to examine the effect of progesterone on lung fibrosis in a mouse model of systemic sclerosis.

**Methods:**

Mice with bleomycin-induced lung fibrosis treated with progesterone subcutaneously for 21 and 28 days. Blood was collected for hormone and cytokine measurement at the end of treatment then, skin and lung tissues were harvested for histological assessment, gene expression, cytokine, hydroxyproline, and gelatinase measurement.

**Results:**

Trichrome staining and hydroxyproline measurements showed that progesterone treatment increased the content of collagen in fibrotic and normal lung tissues. Progesterone increased α-SMA (P < 0.01), TGF- β (P < 0.05) and decreased MMP9 (P < 0.05) in fibrotic lung tissues. Also progesterone treatment decreased the gene expression of *Col1a2* (P <0.05), *Ctgf* (P <01), *End1* (0.001) in bleomycin- injured lung tissues. The serum level of TNF-α was decreased, but the serum level of cortisol was increased by progesterone treatment in fibrotic mice (P< 0.05).

**Conclusion:**

Our results showed that progesterone aggravates lung fibrosis in a mouse model of systemic sclerosis.

## Introduction

Systemic sclerosis (SSc) is a heterogeneous autoimmune disease with inflammatory – fibrotic changes in skin and internal organs, especially lungs (1). Lung fibrosis and hypertension are the main causes of morbidity and mortality in SSc (2). Despite the diversity of fibrotic disease and initiating molecular pathways, the biochemical and cellular mechanisms that lead to the fibrosis process are stereotypical. Cell injury and death provoke immune system activation, production of cytokines and growth factors such as TGF-β and CTGF (connective tissue growth factor), and finally transdifferentiation of different cell types to myofibroblasts. As fundamental elements in fibrosis, myofibroblasts produce extracellular matrix (ECM), especially collagen type 1 and matrix metalloproteinases (MMPs). An imbalance between ECM production and degradation causes excessive deposition of ECM and fibrosis in organs (1).

Systemic sclerosis is a female predominate autoimmune disease (3, 4). Women have more robust immune responses to infections and vaccinations at the cost of increased prevalence of autoimmune diseases, but the reasons for these events are not clear. Gender-related factors such as sex-dependent genetic variations and sex hormones contribute to the immune system’s development and responses. Both parenchymal and immune system cells bear receptors for sex hormones and respond to hormonal inputs (5). Progesterone (P) is the primary hormone of the luteal phase and pregnancy, and it influences autoimmune diseases through action on the immune system and parenchymal cells. We investigated progesterone’s effect on cell apoptosis in our previous work because cell injury and death are the primary mechanisms involved in the fibrosis cascade (6).

In the following study, we evaluate progesterone’s impact on other molecular mechanisms of fibrosis in a systemic sclerosis mouse model.

## Material and Methods

### Animal Model of Systemic Sclerosis and Progesterone Treatment

Six to eight week- old female BALB/c mice (weighing about 20 gram), after temporary accommodation to the laboratory conditions were randomly divided into six groups each containig ten mice as follows:

1) Control group (PBS) 2) Bleomycin (BLM) 3) Bleomycin + Progesterone for 21 days (BLM+P21) 4) Bleomycin + Progesterone for 28 days (BLM+P28) 5) Progesterone for 21 days (P21) 6) Progesterone for 28 days (P28).

All animal experiments were approved by Animal Care Committee from Iran University of Medical Sciences (93-03-30-25094).

Bleomycin (BLM) (Nippon Kayaku, Tokyo, Japan) was dissolved in PBS (Phosphate buffered saline) at a concentration of 1.5 U/ml from which 0.075 U/g was injected subcutaneously into the shaved back skin of mice daily for four weeks (7). Three groups include BLM, BLM+P21 and BLM+P28 received bleomycin for 28 days, from which the group BLM or systemic sclerosis (SSc) model group received only bleomycin. In order to study the effect of progesterone (P), mice received 1 mg Progesterone (50 μg/g, pregnancy maintenance dose) subcutaneously daily (8). Progesterone injection was started simultaneously with bleomycin injection in group BLM+P28, but in group BLM+P21, progesterone injection was started after initiation of the inflammatory phase, followed by the initiation of fibrotic phase (one week after initiation of bleomycin injection) on day eight. There were three control groups, groups P21 and P28 received subcutaneous progesterone for 21 and 28 days, respectively, and the PBS or Control group received daily 100μL PBS subcutaneously. Progesterone level in all progesterone receiving groups was between 100 - 60 ng/ml and Estradiol was below 2.5 pg/ml.

### Tissue Harvesting, Histology, and Immunohistochemistry

At the end of the study (day 29), lungs and injured skins were removed under deep anesthesia. The upper lobes of the right lungs were excised for measurement of TGF-β, isolation of RNA, zymography, and lower lobe for hydroxyproline assessment. The left lungs and half of the excised injured skins were fixed in 10% neutral formalin for pathological assessment, and other excised tissues were fixed in liquid nitrogen. Paraffin-embedded tissue slides were stained with Hematoxylin and eosin for morphological evaluation and with Masson’s trichrome to assess the degree of fibrosis. Quantification of pulmonary fibrosis was determined as described in our previous work (6). The immunohistochemistry detection of Alpha-smooth muscle actin (α –SMA) was performed according to our previous work (9) with conjugated primary antibody α-SMA-HRP (1:500, Santa Cruz Biotechnology, Santa Cruz, CA). Percentage of α –SMA positive areas in lung tissues were calculated by imageJ software. Two independent observers performed microscopic tissue evaluations. 

### Hydroxyproline Assay

Hydroxyproline was measured with a hydroxyproline assay kit (Sigma Aldrich, St. Louis, MO, USA) according to manufacturer protocol. Briefly, stored tissues were weighed, homogenized in distilled water and digested overnight in 6N HCl at 120°C (10mg tissues+ 100 μL water + 100 μL 6N HCl). 0.5 mg of tissue hydrolysis and serial concentration of hydroxyproline were transferred to a 96 well plate and evaporated in 60°C oven. Then Chloramine T/Oxidation buffer was added to each tissue samples and standards and incubated at room at room temperature for 5 minutes. Diluted DMAB reagent (50 µl of DMAB plus 50 µl of perchloric acid/isopropanol solution) was added to the wells and incubated at 60°C for 90 min. The absorbance was measured at 560 nm. All obtained data were subtracted from the blank absorbance. Standard curve was plotted using the appropriate values of hydroxyproline standards. Then hydroxyproline amount of tissue samples were determined from the standard curve.

### Blood Sampling and Measurement of Serum Cortisol and TNF- α Concentrations

On day 29, blood samples were obtained from the tail vein and retro-orbital plexus. Blood was incubated at room temperature for 20 minutes, then centrifuged, and serum was separated from the clot. The level of cortisol hormone was measured with standard clinical chemistry analysis on a Roche Cobase411 analyzer. The level of TNF – α protein in the sera was measured with a commercial ELISA Kit (MABTECH AB, Sweden) according to the manufacturer’s instructions.

### TGF-β Measurement in Lung Tissues

Harvested tissues were homogenized in ice-cold buffer (25 mM Tris – Hcl PH= 7.5, 100 mM NaCl, 1% Triton X -100, and Protease inhibitor) and centrifuged at 10,000×*g* for 10 min at 4°C. According to the manufacturer’s instructions, the concentration of TGF-β in the supernatants was measured by ELISA Kit (R&D).

### Gene Expression Measurement

Total RNA from Lung tissue was extracted using “RNX-Plus” (Sinaclon, Iran), then digested with Recombinant Dnase1 (Takara, Japan) and characterized for quantity and integrity. Complementary DNA (cDNA) was synthesized using PrimeScript RT reagent Kit (Takara, Japan). Gene expression was assessed by SYBR-Green-based real-time PCR by using SYBR-Green master mix (Takara, Japan) protocol and amplification thermocycler machine (Rotor Gene-Q, Germany). The gene-specific primers were listed in **Table 1**, and the β-glucuronidase gene (*Gusb*) was used to normalize the qPCR data. The specificity of the primers was determined by melting curve analysis and agarose gel electrophoresis. The obtained threshold cycle values (Ct) were normalized with the means of *Gusb*, and the normalized values were calculated according to the 2^−ΔΔCT^ method for each animal.

**Table 1:** The primer list used for real time PCR.

Official Symbol	NCBI Reference Sequence (RefSeq)	Forward primer	Reverse primer
**Col1a1**	NM_007742.4	AGCACGTCTGGTTTGGAGAG	GACATTAGGCGCAGGAAGGT
**Col1a2**	NM_007743.3	CCAGCGAAGAACTCATACAGC	GGACACCCCTTCTACGTTGT
**Edn1**	NM_010104.3	CTACGAAGGTTGGAGGCCAT	TGGGGGAGCTCTGTAGTCAA
**Ctgf**	NM_010104.4	AGACCTGTGCCTGCCATTAC	ACGCCATGTCTCCGTACATC
**Gusb**	NM_010368.1	GCTCGGGGCAAATTCCTTTC	CTGAGGTAGCACAATGCCCA

### Gelatin Zymography

Zymography was done according to our previous work (9). Briefly, The excised lung tissues for zymography were homogenized in ice-cold homogenization buffer (25 mM Tris–HCl PH 7.5, 100 mM NaCl, 1% Triton X -100 and Protease inhibitor) and centrifuged at 10,000×g for 10 min at +4°C. The supernatants were stored at −40°C until further analysis. 20 μg of total protein was electrophoretically (constant voltage125) separated in SDS polyacrylamide (10%) gel containing 1 mg/ml gelatin. Gels were then treated with 2.5% Triton X-100 for 45 min at room temperature to remove SDS. Zymograms were subsequently incubated 16 hours at 37°C in developing buffer (50 mM Tris-HCl, pH 7.8, 0.2 M NaCl, 5 mM CaCl2, and 0.02% v/v Triton –X 100). The gels were stained with 0.5% Coomassie Blue R-250 and destained in 10% acetic acid and 30% ethanol in dH2O. Human recombinant MMP9 (R&D) protein was used as positive control. Prestained protein marker (SMOBIO 9-245 kDa) was used as molecular weight standard. For densitometric analysis of bands,imageJ software was used.

### Statistics

All experiments were individually repeated at least twice. Statistical analysis was performed using GraphPad Prism software. Groups greater than two were analyzed with One-Way ANOVA, Tukey,s multiple comparisons test. If conditions were two, an unpaired t-test was used for data analysis. All data were expressed as a mean ± SEM. Differences were considered significant When P < 0.05, 0.01, 0.001 and 0.0001 denoted as *, **, *** and **** respectively. 

## Results

### The Effect of Progesterone onLung Fibrosis

**Figure 1:**
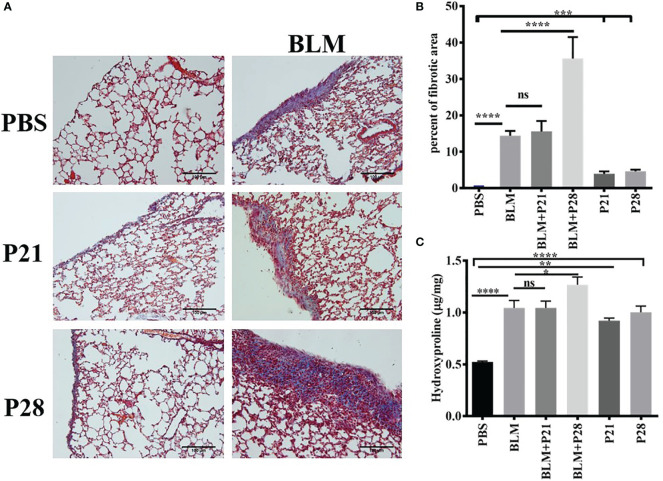
Progesterone aggravates fibrosis in lung tissue. Bleomycin - treated mice received progesterone simultaneously or one week after initiation of bleomycin injection. On day 29 mice were deeply anesthetized and lungs were excised for pathologic and hydroxyproline assessment. **(A)** Masson trichrome staining of the lungs. 4 µm sections of paraffin embedded left lungs were stained with Masson trichrome and examined under light microscope. Original magnification = 200X. Scale = 100 μm **(B)** Quantitative Lung fibrosis. Photographs of ten random fields were taken for each lung slide and then percentage of fibrosis calculated for each lung by ImageJ software. **(C)** Lung hydroxyproline content. Excised lower lobe of right lungs were hydrolyzed with HCl then hydroxyproline contents were measured in tissue hydrolysis. Results represented as mean ± SEM, n = 10 mice in each group. Data were analyzed with One – way ANOVA, Tukey multiple comparisons test. BLM, Bleomycin; P, Progesterone; ns, non - significant, **** = P < 0.0001, *** = P < 0.001, ** = P < 0.01, * = P < 0.05.

Co-administration of progesterone and bleomycin for four weeks in group BLM+P28 increased collagen deposition considerably compared to systemic sclerosis (SSc) model group who received bleomycin alone (p <0.0001). Administration of progesterone for 21 days in group BLM+P21 did not change the amount of fibrosis significantly. Progesterone reception alone for 21 and 28 days caused significant subpleural fibrosis in groups P21 and P28 compared with the Control (PBS) group (P <0.001) (**Figures 1A, B**).

Co-administration of P (progesterone) and bleomycin for 28 days in goup BLM+P28 enhanced hydroxyproline amount in lung tissues as compared to Bleomycin group (P<0.05), however progesterone treatment for 21 days in group BLM+P21 did not change significantly the amount of hydroxyprolinein fibrotic lung tissues. Treatment of mice with progesterone alone increased the amount of hydroxyproline significantly compared with the Control group (P < 0.01 in P21 days and P < 0.0001 in P28 days) (**Figure 1C**).

### The Effect of Progesterone on Myofibroblasts in the Lung Tissue

**Figure 2:**
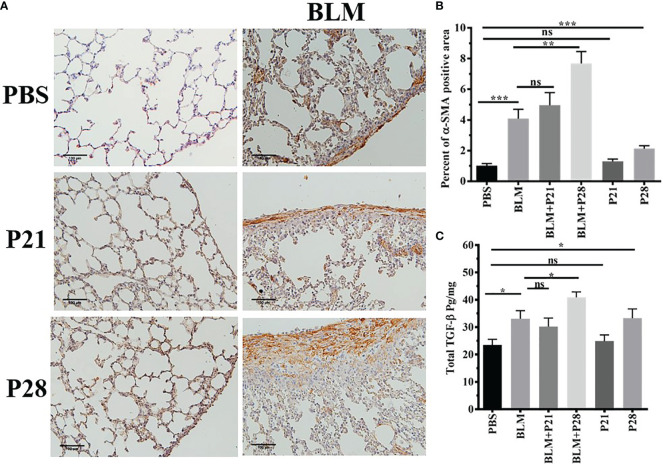
Progesterone increases expression of α-SMA and TGF-β in fibrotic lung tissue. **(A)** 5 µm sections of paraffin embedded left lungs were taken. Slides incubated with anti α-SMA antibody, stained with Diaminobenzidine and counter - stained with Haematoxylin. Original magnification = 200X. Scale = 100 μm **(B)** Quantitative expression of α-SMA in lung tissues. **(C)** TGF-β in lung tissues. Results are represented as mean ± SEM, n= 5 - 10 mice in each group. Data were analysed with One –way ANOVA, Tukey multiple comparisons test. BLM, Bleomycin; P, Progesterone; ns, non -significant, *** = P < 0.001, ** = P < 0.01, * = P < 0.05.

The most abundant expression of α-SMA was shown in mice who received bleomycin plus progesterone for 28 days (BLM+P28). Therefore, 28 days’ administration of progesterone with BLM had a synergistic effect in the expression of α-SMA (P <0.01). Progesterone treatment for 21 days in group BLM+P21 did not change the amount of α-SMA significantly. Progesterone reception alone for 28 days in group P28 increased α-SMA compared to the Control group (P < 0.001) (**Figures 2A, B**). 

### The Effect of Progesterone on TGF-β in Lung Tissue

Bleomycin injection increased lung tissue TGF-β in parallel with fibrosis. TGF-β in fibrotic lung tissue (BLM) was statistically elevated compared to the Control group (P < 0.05). There was no statistical difference between BLM and BLM+P21 groups. Progesterone injection for 28 days in group BLM+P28 augmented the amount of TGF-β in the lungs of fibrotic mice in comparison to the BLM group (P < 0.05). Whereas injection of progesterone for 21 days (P21) did not alter the amount of TGF-β in the healthy lung tissue, 28 days injection of progesterone in group P28 significantly increased the amount of this cytokine (P < 0.05) (**Figure 2C**).

### The Effect of Progesterone on Relative Gene Expression of Collagen I, *Ctgf*, and *End1* in Lung Tissue

To examine the effects of progesterone on gene expression, total mRNA from lung tissue was analyzed by real-time PCR and normalized with a reference gene, Beta- glucuronidase (*Gusb*).

**Figure 3:**
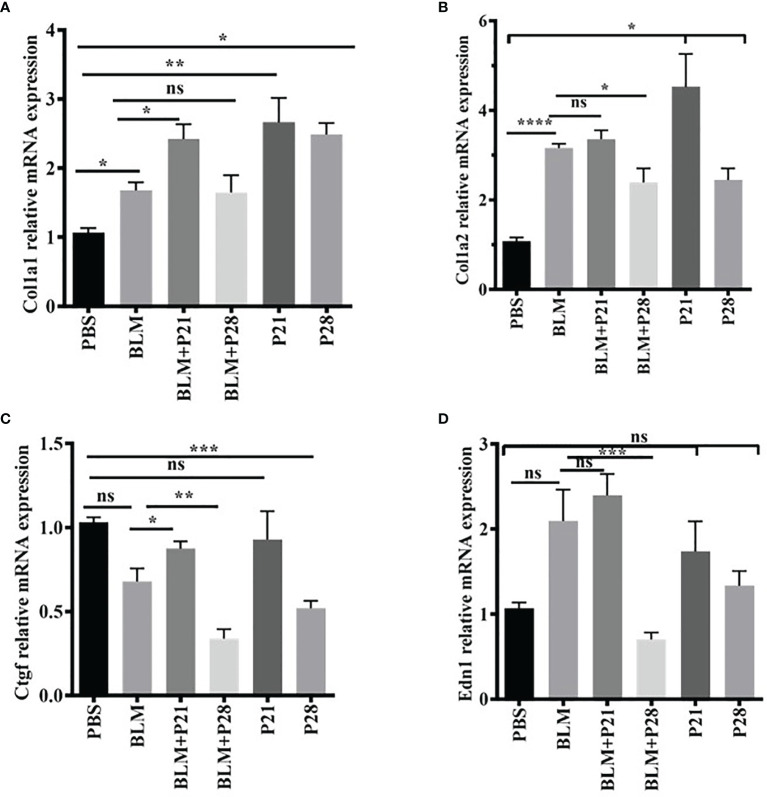
The effect of progesterone on relative gene expression of *Col1a1*, *Col1a2*, *Ctgf*, *Edn1*. Total RNA was isolated from lung tissues, cDNA was synthesized and quantified by real – time PCR. **(A)** Relative expression of *Col1a1* in lung tissue. **(B)** Relative expression of *Col1a2* in lung tissue. **(C)** Relative expression of *Ctgf* in lung tissue. **(D)** Relative expression of *Edn1* in lung tissue. Results are represented as mean ± SEM, n = 5 mice in each group. Data were analyzed with One – way ANOVA, Tukey multiple comparisons test. BLM, Bleomycin; P, Progesterone; ns, non - significant, **** = P < 0.0001, *** = P < 0.001, ** = P < 0.01, * = P < 0.05.

Relative gene expression of collagen type one alpha helixes (*Col1a1* and *Col1a2*) were elevated in BLM received group compared with the Control group (P < 0.05, P < 0.0001, respectively). Progesterone increased mRNA expression of *Col1*alfha chains until day 21 in both fibrotic and normal lung tissues; however, after 21 days, the expression of this gene was relatively reduced. Progesterone significantly increased *Col1a1* in BLM+ P21 group compared with the BLM group (P < 0.05), however, this hormone decreased gene expression of *Col1a2* in the BLM+P28 group compared with BLM group (P < 0.05) (**Figures 3A, B**). Administration of P alone for 21 and 28 days augmented the gene expression of *Col1a1* and *Col1a2* in normal lung tissue compared with the PBS control group (P < 0.05) (**Figures 3A, B**).

BLM injection could not increase the connective tissue growth factor (Ctgf) mRNA in fibrotic lung tissues. Twenty-eight days of injection of progesterone significantly decreased the *Ctgf* gene expression in fibrotic and healthy lung tissue. Therefore, the relative mRNA expression of *Ctgf* abrogated in the BLM+P28 group compared with the BLM group (P < 0.01) and in P28 compared with the PBS group (P < 0.001) (**Figure 3C**).

BLM injection increased mRNA of endothelin-1 (*Edn1*) in a non-significant manner compared with the PBS group. Twenty-eight days of injection of progesterone decreased mRNA expression of *Edn1* in fibrotic and healthy lung tissue; however, this decrement was only significant in the group of BLM+P28 as compared with the BLM group (P < 0.01) (**Figure 3D**).

### The Effect of Progesterone on the Amount and Activity of Gelatinases

**Figure 4:**
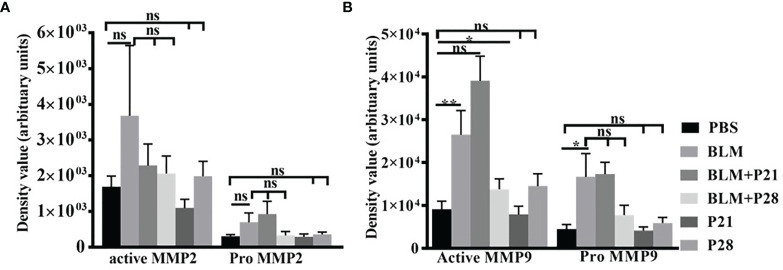
The effect of Progesterone on density values of gelatinases in lung tissue. Lung tissues were processed for gelatine zymography then densitometric analyses of zymograms were done by image J software. **(A)** Density values of MMP2. **(B)** Density values of MMP9. Results represented as mean ± SEM, n = 8-10 mice in each group. Data were analyzed with One – way ANOVA, Tukey multiple comparisons test. BLM, Bleomycin; P, Progesterone; MMP, matrix metalloproteinase; ns, non-significant, ** = P < 0.01, * = P < 0.05.

Analysis of zymograms showed that progesterone injection in group BLM+P21 intensified all four bands of gelatinases in a non-significant manner compared to the BLM group (**Figures 4A, B**). Co-administration of bleomycin with progesterone for 28 days abrogated MMP9 and MMP2 bands in zymograms. Therefore, active MMP9 in BLM+P28 was significantly deduced compared with the BLM group (P < 0.05), but a reduction in pro MMP9, pro, and active MMP2 was not significant. Progesterone alone in groups P21 and P28 did not alter the amount of gelatinases in healthy lung tissues (**Figures 4A, B**).

### The Effect of Progesterone on Serum Level of TNF-α and Cortisol

**Figure 5:**
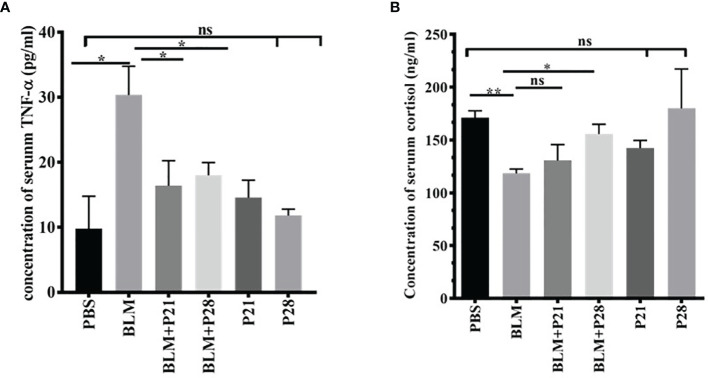
Progesterone is an anti-inflammatory agent. Blood was collected on day 29 and sera were separated for measurements. **(A)** Concentration of serum TNF-α. **(B)** Concentration of serum cortisol. Results are represented as mean ± SEM, n = 5 mice in each group. Data was analysed with One – way ANOVA, Tukey multiple comparisons test. BLM, Bleomycin; P, Progesterone; ns, non - significant, ** = P < 0.01, * = P < 0.05.

For understanding the anti-inflammatory effect of progesterone, two inflammatory markers were assessed in the sera of mice. Bleomycin administration induced a surge in the concentration of serum TNF-α compared with the Control group (P<0.01). Progesterone administration in BLM received groups caused a decline of serum TNF-α; therefore, serum TNF- α in BLM+P groups showed a significantly lower level than the BLM group (p <0.05). In progesterone-only received mice (P21 and P28 groups), there were no significant changes in the serum level of TNF-α (**Figure 5A**).

As shown in **Figure 5B**, the mean serum cortisol levels in Control animals were 171.1 ± 6.57. Administration of Bleomycin for four weeks caused a significant reduction of serum cortisol level (P<0.01). Co-administration of bleomycin and progesterone partially restored the cortisol level in sera (P< 0.05 in BLM+P28). In progesterone-only received mice (P21 and P28 groups), no significant change of cortisol level in sera was detected (**Figure 5B**). 

## Discussion

Our results for the first time showed that progesterone at pregnancy-related physiological concentrations could exacerbate fibrosis in a mouse model of systemic sclerosis.

The results of our study are summarized in **Table 2**, which includes some new findings concerning the bleomycin mouse model (column 1), the effect of progesterone on bleomycin-induced lung fibrosis (column 2,3), and the input of progesterone in normal lung tissues (column 4,5).

**Table 2:** Results of the study.

	BLM To Control	BLM+P21 To BLM	BLM+P28 To BLM	P21 To Control	P28 To Control
***Col1a1***	↑	↑	↔	↑	↑
***Col1a2***	↑	↔	↓	↑	↑
**α- SMA**	↑	↔	↑	↔	↑
**TGF-β**	↑	↔	↑	↔	↑
***Ctgf***	↔	↑	↓	↔	↓
***Edn1***	↔	↔	↓	↔	↔
**Mast cell**	↔	↔	↔	↔	↔
**MMP9**	↑^⁎^	↔	↓	↔	↔
**MMP2**	↔^⁎^	↔	↔	↔	↔
**TNF-α**	↑	↓	↓	↔	↔
**Cortisol**	↓	↔	↑	↔	↔
**Collagen**	↑	↔	↑	↑	↑
**Apoptosis^⁎^**	↑	↔	↔	↔	↔
**Fibrosis**	↑	↔	↑	↑	↑

P, Progesterone; BLM, Bleomycin; Col1a1, Collagen Type I Alpha 1 Chain gene; Col1a2, Collagen Type I Alpha 2 Chain gene; α-SMA, Alpha smooth muscle actin; Ctgf, connective tissue growth factor gene; Edn1, Endothelin -1 gene; MMP9, Matrix metalloproteinase 9; MMP2, Matrix metalloproteinase 2. ↑ = increased, ↓ = decreased, ↔ = does not alter. ⁎ = results adapted from references (4) and (5).

**Figure 6:**
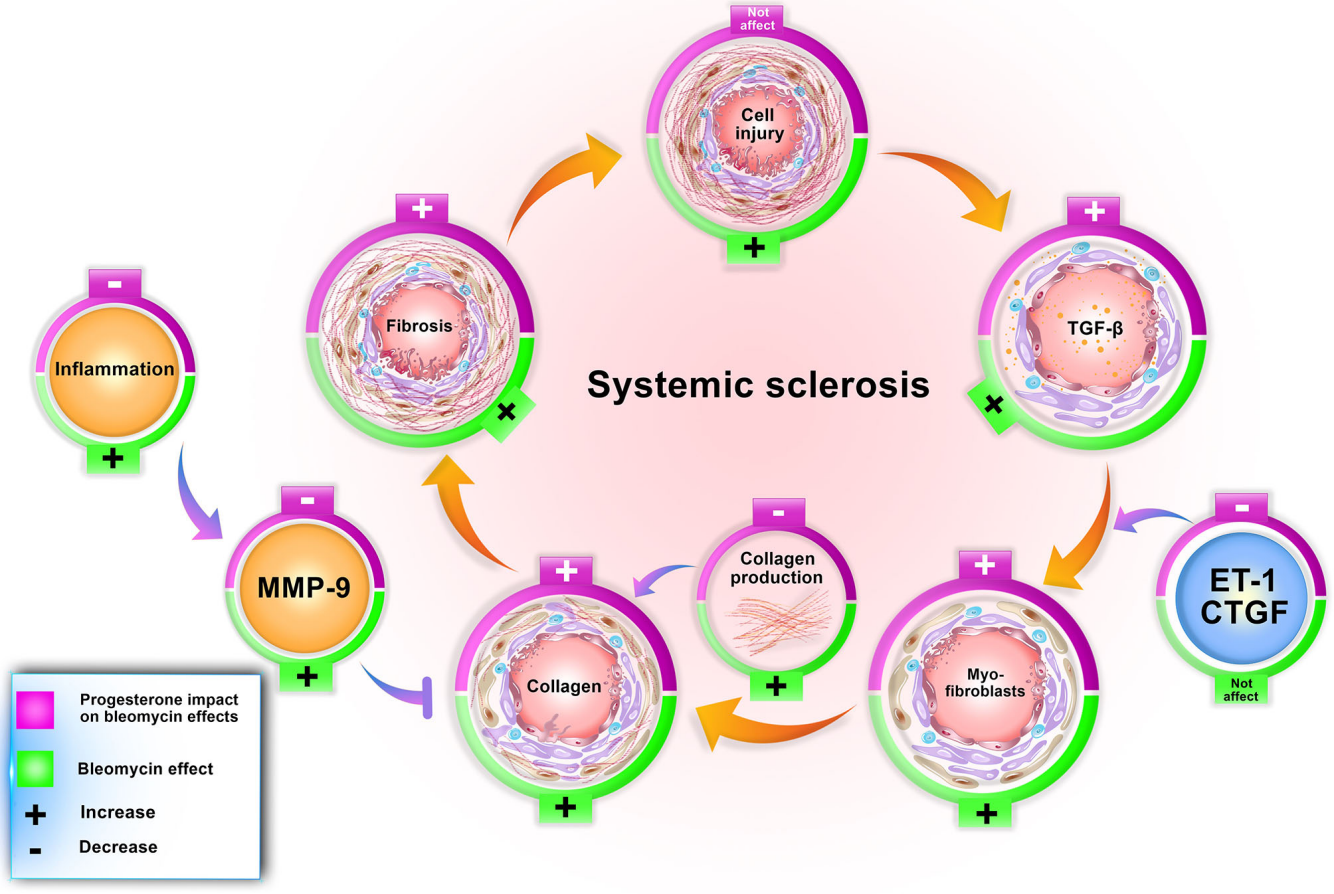
Progesterone aggravates lung fibrosis in bleomycin - induced systemic sclerosis model. Progesterone by increment of the TGF-β, myofibroblasts, and decrement of MMP9 and inflammation augments collagen content in fibrotic lung tissues, therefore aggravates fibrosis. ET, Endothelin; CTGF, connective tissue growth factor; MMP, matrix metalloproteinase.

Our findings confirm other achieved information regarding the bleomycin model. In this model, bleomycin injection induced stereotypical fibrotic molecular cycle (**Figure 6**). Bleomycin induces cellular apoptosis in lung tissue (6), inflammation in the lung and BAL (bronchoalveolar lavage) (10), increases myofibroblasts, TGF-β, and finally, collagen deposition (gene and protein expression), which promotes fibrosis in this tissue. Bleomycin increased MMP9 in lung tissue (9); however, in this method, CTGF and ET-1 did not alter in BALB/c mice (**Table 2**). Subcutaneous administration of bleomycin (BLM) for four weeks caused pulmonary apoptosis (6) and fibrosis which was started from the pleura and subpleural area and extended to the lung parenchyma compatible with the most apoptotic areas (6). On the other hand, in this model, central areas of the lung were not affected. The same pattern of initiation and progression of fibrosis was seen in interstitial lung disease of systemic sclerosis and resembled human lung fibrosis in SSc (2, 6, 7).

In control groups, progesterone augmented gene and protein expression of collagen I in normal lung tissues, while the amount of TGF-β and myofibroblasts did not alter until the 21^st^ day of progesterone administration. However, with increasing progesterone administration from day 21 to day 28, due to unknown etiology, the number of myofibroblasts and TGF-β increased in normal lung tissues (**Table 2**). This finding indicates that the repair or fibrosis process starts with increasing the days of progesterone administration. Progesterone without influencing cell injury and apoptosis (6) induced repair factors in normal lung tissues.

Progesterone in BLM injured lung tissue did not alter the cell apoptosis (4) but increased the number of myofibroblasts and TGF-β in this tissue. Progesterone in BLM injured mice decreased MMP9 in lung tissue, inflammatory cells in BAL (10), and TNF-α in serum with a simultaneous increment of serum cortisol. These progesterone effects caused extracellular matrix (ECM) expansion in mice who received bleomycin plus progesterone for 28 days (**Figure 6**).

Myofibroblasts are activated fibroblasts with α-SMA fiber in their cytoplasm. These cells appear transiently during the wound healing process and are removed by apoptosis after the process is completed, while in pathological fibrosis, these cells persist in the fibrotic tissues (11). In accordance with previous studies, in our investigation, the number of myofibroblasts increased in parallel with fibrosis progression, and the highest expression of α-SMA was indicated in the group with the most severe lung fibrosis, namely the BLM+P28 group. Progesterone increased myofibroblasts in normal and fibrotic lung tissues. Progesterone activates and proliferates liver fibroblasts (12) but inhibits heart fibroblasts’ proliferation (13). Progesterone inhibits the proliferation of fibroblasts in the skin but causes activation and production of collagen type one and MMP1 by these cells (14). The conflicting finding concerning progesterone’s effect on fibroblasts could be due to the heterogeneity of fibroblasts, different microenvironment in tissues, or different study methods.

TGF-β is the cardinal cytokine in fibrosis development; the cooperative interaction of TGF-β with CTGF and ET-1 aggravates the fibrosis process (15). We detected the highest concentration of TGF-β in groups with the highest amount of fibrosis (BLM+P28 group). However, in this study, CTGF and ET-1 did not increase in fibrotic groups; even CTGF was decreased in the presence of progesterone in BLM+P28 group. In a study by Lasky et al. the amount of CTGF and ET-1 did not increase in bleomycin-treated BALB/c mice, which can explain the relative resistance of BALB/c mouse strain to the induction of fibrosis (16, 17). Several investigations showed that the increment of CTGF and ET-1 expression required the activation of smad1 (mothers against decapentaplegic homolog 1) and ERK1 (extracellular signal-regulated kinase 1) through TGF-β receptors I, II, and ALK1 (18–20). These signaling pathways must be further evaluated in BALB/c mice for understanding the involved mechanisms in the production of CTGF and ET-1. In a recent study by Kunzmann et al. Progesterone dose-dependently inhibited TGF-β activated Smad signaling and suppressed TGF-β1/Smad-induced upregulation of CTGF, transgelin, and plasminogen activator inhibitor-1 (PAI-1) in lung epithelial cells (21). Progesterone administration in 28 days increased TGF-β and decreased CTGF in normal and fibrotic lung tissues. Progesterone’s inhibition of Smad signaling can explain why increased TGF-β cannot induce CTGF production in progesterone received mice, but further investigation needs in this regard.

We previously demonstrated the increased gelatinases (MMP2 and MMP9) activity in bleomycin-induced systemic sclerosis, and MMP9 was the main gelatinase in the lung tissues (9). In this study, progesterone does not alter MMP9 in 21 days of administration; however, progesterone administration for 28 days decreased the amount of MMP9 in fibrotic lung tissues. Interactions between progesterone, TGF-β, and MMPs are complex and may be different in various tissues. Progesterone is declared as a repressor of MMPs (22–24); this hormone by reduction of MMP9 and dephosphorylation of focal adhesion kinase (FAK) in lung adenocarcinoma cells inhibits migration and invasion of lung cancer cells (25). In endometrial tissue, progesterone increased gene and protein expression of TGF-β1, and this cytokine in negative feedback downregulates progesterone receptors. TGF-β has different effects on MMPs; it reduces matrilysin (MMP7) but augments gelatinases (MMP2, MMP9) in endometrial tissues. Progesterone reduced TGF- β mediated increment in gelatinases; thus, declining progesterone levels may result in loss of progesterone’s inhibitory effect on TGF- β mediated up-regulation of MMPs. After the mid-luteal phase in the uterine, progesterone level and action decreased, which would cause upregulation of proteases, the permeability of vessels, entrance of the inflammatory cells into the uterine, and production of inflammatory cytokines such as TNF-α. These events cause more activation of MMPs and, finally, loss of endometrial tissue (26–29).

The mentioned scenario concerning progesterone’s action in endometrial tissue is repeated by progesterone in fibrotic lung tissues. Bleomycin injection augments TGF-β in lung tissues then this cytokine escalates gelatinases (9). Progesterone injection in fibrotic mice further raises TGF-β level but declines the amount of MMP9. On the other hand, progesterone as an anti-inflammatory agent (30) in fibrotic mice with an increment of serum cortisol and decrement of TNF-α diminishes inflammation, reducing the activation of MMPs. MMPs have a significant role in ECM homeostasis; hence, reducing their function prevented ECM and collagen degradation (31). As a result, progesterone in fibrotic lung tissues with boosting myofibroblasts, TGF-β, and remitting MMPs induces ECM deposition and fibrosis (**Figure 6**).

Gharaee- Kermani et al. indicated the reduction of lung fibrosis in ovariectomized rats and replacement of Estradiol returned the lung collagen deposition and fibrotic changes (32). Our obtained results, incoherent with this study about the estrogen effect on lung fibrosis, demonstrate that female hormones (estrogen and progesterone) can exacerbate lung fibrosis in susceptible women. This event may occur in susceptible women during pregnancy, in which serum and tissue levels of female hormones reach their highest concentration. Clinical studies concerning the influence of pregnancy in women with systemic sclerosis emphasize that pregnancy in the early course of diffuse SSc increased the risk of developing cardiopulmonary and renal problems. Pregnancy appears to exacerbate organ involvement or adversely affects the 10-year survival of scleroderma patients (33).

It is known that progesterone is a fibrotic agent in the liver (34). Progesterone activates rat hepatic stellate cells (the primary cell type involved in liver fibrosis), and in a dose-dependent manner, enhances ROS generation and TGF-β1 expression by these cells while acting oppositely to the favorable effects of Estradiol (12). In heart tissue, 17β-estradiol, its metabolites, and progesterone inhibit rat cardiac fibroblasts’ growth in a gender-independent fashion (13). Progesterone, with producing repair factors such as TGF-β and amphiregulin in the lungs, reduces alveolar penetration of proteins and decreases influenza infection mortality (35). Also, this hormone with an anti-inflammatory effect reduces mortality in COVID – 19 infections (36). Progesterone repairs myelin fibers in the central nervous system and inhibits the progression of multiple sclerosis (37). In a simple review of these experiments, progesterone increases tissues’ abilities for healing through anti-inflammatory action or repairing process. However, the discrepancy between various effects of progesterone can be due to the tissue pathology. Progesterone reduces mortality in severe infections by accelerating the repair process, prepares uterine cavity for embryonic implantation, increases fibrosis in the fibrotic liver and lungs, and is a beneficial factor in heart and brain ischemia.

This study cannot explain why progesterone has a dual effect in fibrotic and normal lung tissue in the expression of collagen mRNA. It is unknown whether progesterone in negative feedback control mechanisms, such as downregulation of progesterone receptors and affecting collagen mRNA expression, regulates collagen content in tissues. Also, it is recommended to analyze the fibrotic factors and progesterone receptors in time courses to understand progesterone action mechanisms better.

## Conclusion

In conclusion, our study showed progesterone aggravates fibrosis in bleomycin-induced systemic sclerosis.

## Data Availability Statement

The original contributions presented in the study are included in the article/supplementary material. Further inquiries can be directed to the corresponding author.

## Ethics Statement

The animal study was reviewed and approved by Animal Care Committee from Iran University of Medical Sciences. 

## Author Contributions

FV, AH, SP and NM carried out the experiments, FV, KM, and NM interpreted the data and wrote the manuscript, and FV, KM, HP, and NM conceived and designed the study. All authors contributed to the article and approved the submitted version. 

## Funding

This study was supported financially by Iran University of Medical Sciences under the grant No. (93–03–30–25094).

## Conflict of Interest

The authors declare that the research was conducted in the absence of any commercial or financial relationships that could be construed as a potential conflict of interest.

## Publisher’s Note

All claims expressed in this article are solely those of the authors and do not necessarily represent those of their affiliated organizations, or those of the publisher, the editors and the reviewers. Any product that may be evaluated in this article, or claim that may be made by its manufacturer, is not guaranteed or endorsed by the publisher.
